# Heterologous expression of NRPS and PKS pathways in *Escherichia coli*: from bottlenecks to biosynthetic platforms

**DOI:** 10.1042/EBC20250044

**Published:** 2026-08-19

**Authors:** Lisa Göbner, Tobias A. M. Gulder, Paul M. D'Agostino

**Affiliations:** 1 Department of Natural Product Biotechnology, Helmholtz Institute for Pharmaceutical Research Saarland (HIPS) and Helmholtz Centre for Infection Research (HZI), Campus E8.1, 66123 Saarbrücken, Germany.; 2 Department of Pharmacy and PharmaScienceHub (PSH), Saarland University, Campus E8.1, 66123 Saarbrücken, Germany.; 3 Chair of Technical Biochemistry, Technical University of Dresden, Bergstr. 66, 01069 Dresden, Germany

**Keywords:** biosynthetic gene cluster, expression host, heterologous expression, natural products

## Abstract

Microbial natural products (NPs) represent one of the most important sources of bioactive compounds in medicine, agriculture, and biotechnology. Non-ribosomal peptide synthetases (NRPSs) and polyketide synthases (PKSs) are large multidomain complexes that enable the efficient generation of highly diverse and complex NPs. Advances in genome sequencing and bioinformatics have revealed an enormous and largely untapped biosynthetic potential encoded in microbial genomes. However, the majority of NPs remain inaccessible due to a lack of genetic tools, silent biosynthetic gene clusters (BGCs) or the inability to culture the vast majority of microbes. Heterologous expression has therefore emerged as a key strategy to access and engineer NPs. Among available expression platforms, *Escherichia coli* is a pivotal host due to its rapid growth, genetic tractability, and unparalleled molecular toolbox. This review focuses on heterologous expression of NRPS/PKS pathways in *E. coli*, with particular emphasis on experimental optimisation strategies. Drawing on selected case studies, we highlight how systematic approaches—such as promoter refactoring, expression-rate tuning, chaperone or helper protein co-expression, metabolic engineering, precursor feeding, and cultivation optimisation—can transform initially unsuccessful systems into productive expression platforms. Collectively, these examples illustrate that apparent limitations in *E. coli* are engineering challenges rather than intrinsic barriers.

## Introduction

Natural products (NPs) have had a profound impact on medicine, agriculture, and their corresponding industries [1]. Owing to their remarkable chemical diversity and biological activities, NPs have yielded many of the most important therapeutics and biotechnological agents in use today, with over half of pharmaceuticals in use being NPs or their derivatives [2,3]. Unlike primary metabolites, which are essential for cellular growth and universally present in dividing cells, NPs (also known as secondary metabolites or specialised metabolites) are not essential for survival but may provide an important ecological advantage such as mediating competition, chemical defence, nutrient acquisition or interspecies interactions [2]. Major classes of microbial NPs include polyketides (PKs) [4], non-ribosomal peptides (NRPs) [5], ribosomally synthesised and post-translationally modified peptides (RiPPs) [6], terpenes [7], and alkaloids [8].

NRPs and PKs are one of the most significant classes of NPs and are distinguished by their modular, assembly-line-like biosynthesis, which enables the programmable incorporation and diversification of building blocks, thereby allowing complex and pharmacologically important metabolites beyond the scope of most other NP classes. NRPs are biosynthesised from a diverse pool of over 300 amino acids, including d-configurated, *N*-methylated, and other unique non-proteinogenic building blocks [5,9], whereas PKs are built of acetate, malonate, and other short-chain carboxylic acid building blocks [10,11]. They are assembled by genetically programmed and ribosome-independent molecular machineries, i.e., non-ribosomal peptide synthetases (NRPSs) and polyketide synthases (PKSs) [12]. In addition, the two systems can be integrated into a single pathway to form hybrid NRPS/PKS-NPs. This biosynthetic compatibility results from the similar biosynthetic logic of NRPS and type I PKS. Both assembly-line-like pathways are organised in a modular fashion, with each module typically responsible for selection, activation and incorporation of a defined building block into the growing NP, although iteration or module skipping can occur. Each module consists of several catalytic domains, each responsible for a specific reaction: selection and activation are mediated by an adenylation (A) domain in NRPS or acyltransferase (AT) domain in PKS, which loads the substrate onto a peptidyl carrier protein (PCP) or acyl carrier protein (ACP) *via* a thioester bond to a post-translationally attached phosphopantetheinyl arm. Chain elongation occurs through peptide bond formation catalysed by condensation (C) domains in NRPS or *C*–*C* bond formation by Claisen condensation-like reactions by ketosynthase (KS) domains in PKSs, resulting in stepwise NP construction along the enzymatic assembly line. Non-compulsory auxiliary domains provide further structural diversity, enabling epimerisation (E), methylation (MT), or cyclisation (Cy) reactions in NRPS systems. In PKS, the β-keto group introduced during elongation can be differentially processed depending on the presence of reducing domains—ketoreductase (KR), dehydratase (DH), and enoyl reductase (ER)—thereby controlling the oxidation state. In both systems, biosynthesis of the core structure is typically terminated by a thioesterase (TE) domain, which catalyses hydrolysis or macrocyclisation. Prior to or after TE-mediated release of the NP, additional modifications can be introduced by tailoring enzymes (e.g., halogenation, hydroxylation, or cross-linking in glycopeptides) [13–15]. NRPSs and PKSs display high sequence conservation due to their modular domain architecture across phylogenetically distant organisms and are typically encoded within discrete biosynthetic gene clusters (BGCs), although multiple and diverse clusters can be present within a single genome [16]. These features have enabled genome-based strategies for NP discovery, which have been fundamentally transformed by the rapid advancement of next-generation sequencing technologies and bioinformatics [17].

Genome mining tools together with curated databases allow systematic identification and annotation of BGCs from both genomes of cultured isolates and/or metagenomes [18–29]. Genome mining has revealed a largely untapped biosynthetic reservoir, particularly among slow-growing organisms and microbes inhabiting extreme environments. However, the ability to predict a BGC does not guarantee access to its corresponding NP. One approach of NP discovery relies on the cultivation of microorganisms followed by organic solvent extraction and isolation of the NP of interest [30]. Only a small fraction—estimated at less than 1%—of microbial species can be readily cultured under laboratory conditions, leaving the vast majority of biosynthetic potential inaccessible by this approach [31]. Furthermore, the majority of BGCs remain poorly expressed or silent under standard laboratory conditions, likely due to tight regulatory control and the metabolic burden associated with NP biosynthesis [2,32]. Strategies to overcome these challenges generally fall into two categories: (i) the activation and/or optimisation of a BGC of interest either by changing physiological parameters (e.g., “One Strain Many Compounds” OSMAC [33]) or by genetic manipulation of the native cultivated organism, or (ii) the transfer of the BGC into a genetically tractable heterologous host followed by recombinant expression [34,35]. The latter approach has emerged as a particularly versatile solution, especially for slow-growing or unculturable organisms, or those that are not amenable to genetic manipulation.

Heterologous expression enables pathway refactoring, controlled expression, metabolic engineering, systematic optimisation, and a defined metabolic background compared with using the native producer. The implementation of heterologous expression requires capture of the target BGC from its native organism and its transfer to the heterologous host of choice. The cloning and assembly of these constructs are technically demanding, although advanced approaches such as linear-linear or linear-circular homologous recombination (LLHR or LCHR) [36], transformation-associated recombination (TAR) [37], direct pathway cloning (DiPaC) [38] or CRISPR-associated methods [39–41] provide viable solutions. Each methodology comes with distinct advantages and disadvantages, such as the size of the BGC that can be captured or the ability to further manipulate the DNA sequences. However, each provides the genetic template necessary for downstream heterologous expression and functional analysis [38,42–45], whereas synthetic DNA offers an increasingly viable alternative when direct DNA isolation proves challenging or infeasible.

Historically, actinomycetes such as the genus *Streptomyces* have been the dominant heterologous hosts for expression of NRPS/PKS BGCs, largely because they are the origin of many clinically important NPs [46]. Different bacterial platforms such as *Bacillus subtilis*, *Pseudomonas putida*, or cyanobacteria have also been explored [46–48]. Eukaryotic hosts, including *Saccharomyces cerevisiae* and filamentous fungi such as *Aspergillus nidulans*, are particularly valuable for plant and fungal NPs due to similar mechanisms of transcription and translation in eukaryotes [46]. However, as the most extensively studied biological system, *Escherichia coli* offers unmatched versatility in genetic and metabolic engineering. Its rapid growth, simple cultivation requirements, well-characterised genome, and vast molecular toolbox of vectors, promoters, strains, and genome engineering technologies make it an exceptionally attractive heterologous host for BGCs that originate from various phyla [46,49–51].

*E. coli* has a long history of routine use for the overproduction and isolation of individual recombinant proteins [52]. In this context, successful expression is typically defined by high yields of soluble protein. While certain strains of *E. coli* do harbour NRPS and/or PKS BGCs, it is not a prolific producer of secondary metabolites, and its use as a heterologous host for NRP and PK biosynthesis presents distinct challenges. Heterologous expression of entire NRPS and PKS BGCs requires coordinated functionality across three layers of cellular organisation, including (i) genetic: recognisable promoters, transcriptional compatibility, long polycistronic operons, and defined codon usage; (ii) protein: correct folding of large multidomain enzymes and appropriate post-translational modifications; and (iii) metabolic: sufficient availability of specialised precursors and cofactors, as well as efficient provision and turnover of intermediates throughout the biosynthetic pathway. These interconnected constraints are not intrinsic barriers but represent challenges that can be systematically addressed.

**Figure 1:**
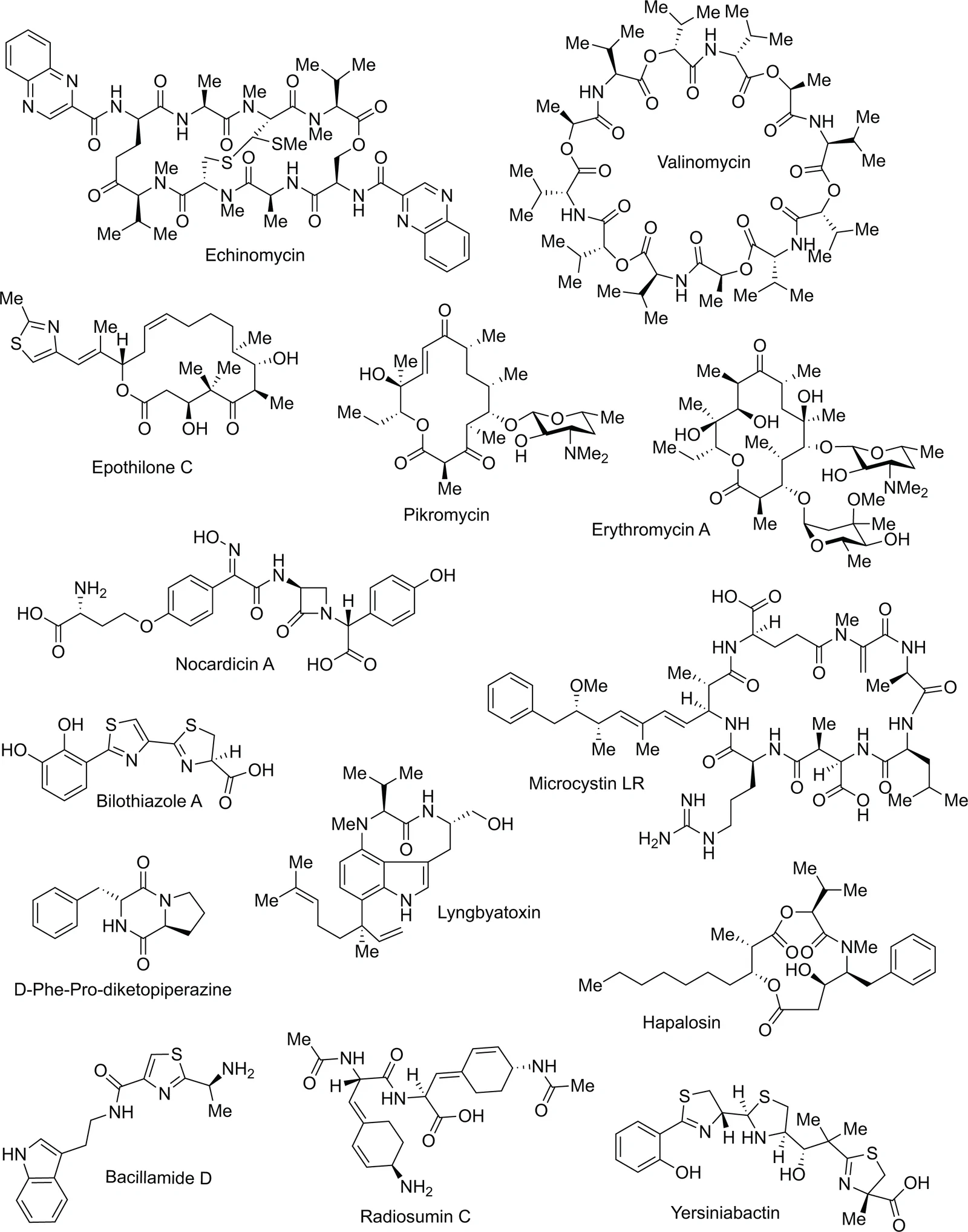
NPs accessed by *E. coli* heterologous hosts. Selected examples of NPs obtained by successful heterologous expressions of NRP/PKs in *E. coli*.

Overall, *E. coli* has evolved from a protein expression workhorse into a flexible and increasingly powerful platform for NP biosynthesis. In the following sections, we discuss heterologous expression of selected NRPS and PKS BGCs in *E. coli* (Figure 1). We highlight how the unique advantages of *E. coli*, combined with practical optimisation strategies, have established it as a central platform for NP heterologous expression for improved NP discovery.

## *E. coli* as a heterologous platform for expression of NRPS and PKS systems

**Figure 2:**
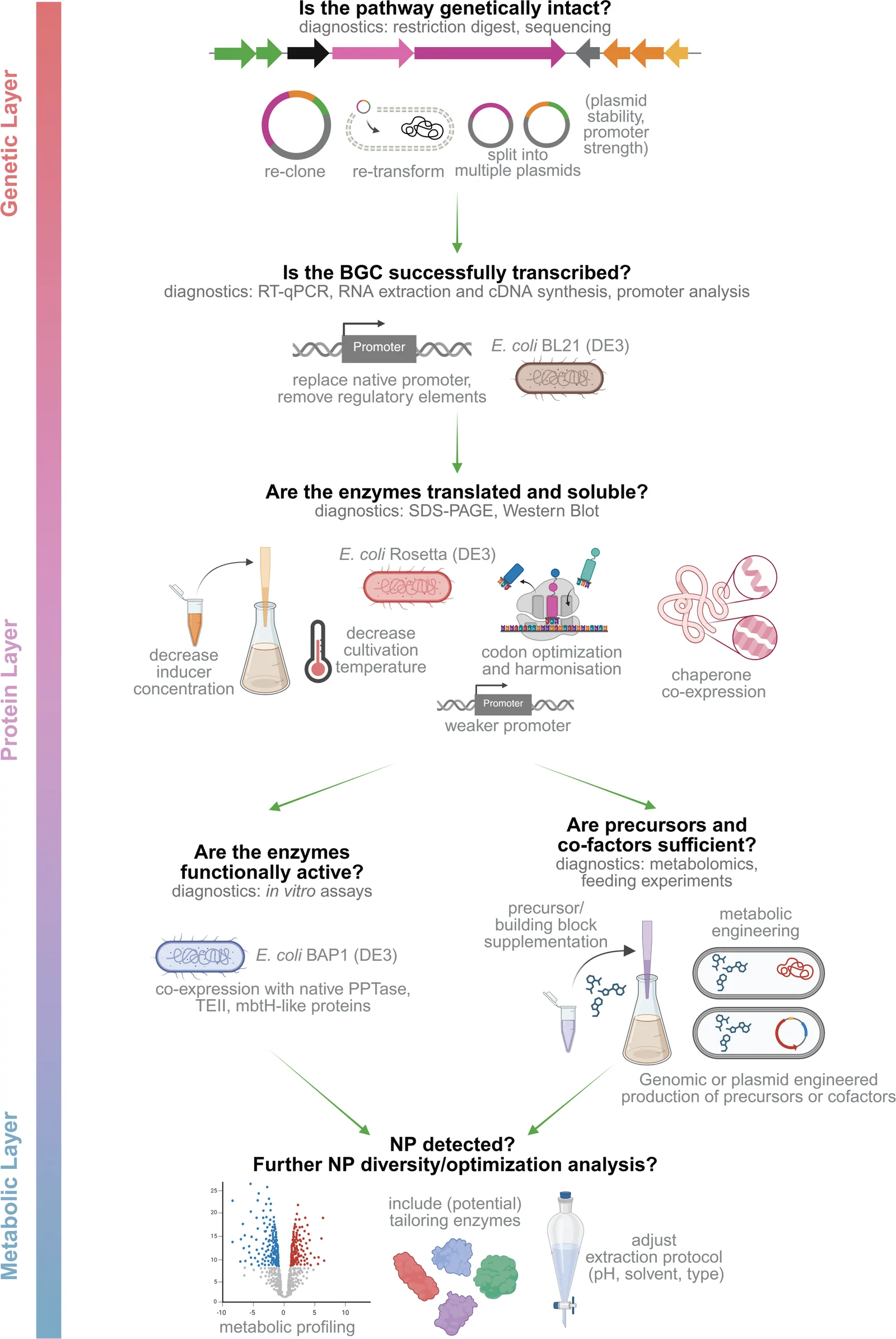
Heterologous expression optimisation workflow. Systematic iterative optimisation workflow across genetic, protein, and metabolic layers for heterologous expression in *E. coli*.

The challenges of heterologous expression at the genetic, protein, and metabolic layers mostly arise from relocating a BGC from its native host, shaped by regulatory and metabolic specificities, into *E. coli*, with the cryptic biosynthesis encoded by most BGCs of interest adding to complexity. A detailed understanding of both native host and the BGC is a helpful first step towards successful heterologous expression. Insights into the native organism, such as GC content, preferred codon usage, and regulatory elements, may inform translational optimisation and overall pathway compatibility. Bioinformatic analyses, such as those provided by BLAST and the conserved domain detection tool [53,54], antiSMASH [27], and the A-domain substrate predictor tool PARAS [55], can provide knowledge of gene function, domain architecture or substrates of the predicted biosynthetic pathway. Such bioinformatic characterisation of the BGC is critical for ensuring compatibility with the *E. coli* heterologous host, thus minimising trial-and-error and increasing the likelihood of success. Consequently, successful pathway reconstitution relies on a structured and iterative optimisation strategy (Figure 2).

### Genetic layer: transcriptional compatibility and expression control

A common barrier to successful NP biosynthesis is transcriptional incompatibility. Successful expression of NRPS or PKS BGCs using native promoters is relatively rare, particularly with phylogenetically distant organisms since they may be poorly recognised by the *E. coli* RNA polymerase and/or transcription factors [56]. Promoter refactoring is therefore a foundational step in many *E. coli*-based expression systems. The two most successful promoters for heterologous expression of NRPS, PKS, or hybrid BGCs in *E. coli* include the strong T7 promoter and the weaker tetracycline-inducible P_tetO_ [57,58].

The T7 promoter originates from the T7 bacteriophage and requires the expression host to harbour the exogenous and highly active T7 RNA polymerase [59]. In a BGC context, the T7 promoter has been successfully used to express NRPS, PKS, and hybrid pathways originating from *Pseudoalteromonas*, including the NRPS BGC responsible for producing the alterochromides [60]. The T7 promoter has a very high transcription rate, but it is limited in mRNA transcript lengths of 20 kb and therefore can be problematic for the expression of large NRPS/PKS BGCs [57]. For this reason, a multi-monocistronic operon approach has been used for the expression of BGCs from some *Streptomyces*, such as for the production of 6-deoxyerythronolide B (6-dEB) and echinomycin [61,62]. Both BGCs were successfully expressed by placing the T7 promoter, RBS, and terminator directly upstream of all biosynthetic genes, enabling balanced expression and improved overall pathway performance [62].

The tetracycline-inducible P_tetO_ is based on the tetracycline resistance cassette from the transposon *Tn10*. The promoter is bidirectional and initiates transcription of the TetR transcriptional repressor in one direction and the BGC of interest in the other. Transcription of the BGC of interest is initiated by addition of tetracycline, which binds to the TetR repressor protein, resulting in a conformational change and dissociation from the DNA [58]. P_tetO_ is very versatile and has proven successful in several organisms, including *P. putida*, *Myxococcus xanthus*, *Streptomyces* spp., and *E. coli*. Heterologous expression of the luminmycins from *Photorhabdus luminescens* DSM15139 was activated by the removal of the repressor TetR, thus resulting in a constitutive promoter [63]. A bi-directional P_tetO_ has also been successful in expression of large BGCs, such as the 55 kb hybrid NRPS/PKS from *Microcystis aeruginosa* PCC 7806 responsible for production of microcystin LR and [D-Asp^3^]microcystin-LR [64]. A P_tetO_ approach has been used in combination with the DiPaC strategy to capture, refactor and heterologously express multiple cyanobacterial NRPS and NRPS/PKS hybrids, including anabaenopeptin [38], hapalosin [65], microginin [66], and the radiosumins [67].

Expression rate tuning can also be achieved through modular control strategies at the pathway level. In the production of d-Phe-Pro-diketopiperazine from *Bacillus brevis* ATCC 8185, a two-plasmid co-expression system with independently inducible promoters outperformed fusion constructs on a single plasmid, as separate up-regulation of the product-releasing module TycB1 alleviated a rate-limiting step [68]. Thus, dividing large BGCs across multiple compatible plasmids for heterologous expression can improve stability, control gene dosage, and be used to adjust expression ratios.

The additional removal of internal regulatory elements such as putative transcriptional terminators within intergenic regions of the BGC is critical during promoter refactoring to ensure complete transcription of the entire operon. Refactoring by removal of transcriptional terminators is showcased with the expression of hapalosin BGC from *Fisherella* sp. PCC 9431 [65] and bacillamide D, a cytotoxin from *Bacillus cereus* DSM 28590 [69]. Successful transcription over the entire operon can be measured by including a fluorescent transcriptional reporter, such as *gfp*, downstream of the BGC. Successful transcription along the entire BGC will result in expression of GFP, as shown for the expression of the volatile organic compound sodorifen from *Serratia plymuthica* WS3236 [70] and the serine protease inhibitor radiosumin from the cyanobacterium *Dolichospermum planctonicum* UHCC 0167 [67]. In cases of failed expression, absence of GFP, or other indications of potential transcriptional disruption, transcriptional analysis can be performed to detect the presence of mRNA. This involves RNA isolation followed by cDNA synthesis and PCR amplification using primers specific to regions spanning the full-length transcript. Successful amplification shows that the mRNA transcription of the respective genes is intact, suggesting that expression failure may lie with translation, biosynthesis and/or metabolism. Absence of PCR products points to absent or incomplete transcription, highlighting the need to alter the promoter or nucleic acid sequence.

Transcriptional challenges are closely linked to translational bottlenecks, as differences in codon usage between the BGC origin and *E. coli* can severely reduce translation efficiency [49,71]. *E. coli* Rosetta and *E. coli* Rosetta2 each harbour a plasmid (pRARE/pRARE2) encoding tRNAs to aid in expression of genes that harbour codons rarely used in *E. coli* [72]. Codon optimisation is another approach to ensure efficient translation of NRPS and PKS genes. This strategy was chosen for the heterologous expression of the *bil* BGC from an uncultured strain of *Bilophila* [73]. Since the BGC was of metagenomic origin and genomic DNA was inaccessible, commercial gene synthesis in combination with codon optimisation was used, which together with mutasynthetic work yielded bilothiazoles A–F. However, codon optimisation may not always be successful and it has been reported that rare codons provide an opportunity for proper protein folding by decreasing the speed of translation [74]. Instead, the recently devised concept of *codon harmonisation* retains the order of rare codons and matches the codon frequency of the targeted host with the codon usage of the native host. The approach successfully activated an unnatural PKS in *E. coli* and is available as an online bioinformatic tool [75].

### Protein layer: folding, activation, and functional assembly

Protein folding represents one of the most critical challenges in *E. coli* expression hosts and can be targeted with temperature-based approaches or co-expression with cellular chaperones. Rapid overexpression frequently leads to misfolding and aggregation, resulting in inactive inclusion bodies [76]. A recurring observation across multiple studies is that slowing down protein synthesis improves solubility and activity. This can be achieved by reducing cultivation temperature (typically to 16–25°C) as demonstrated for the expression of the protein kinase C activator lyngbyatoxin from *Moorea producens* [77]. No production was observed at 30°C but lowering the temperature to 18°C resulted in the production of the respective NP. Co-expression of molecular chaperones such as GroEL/GroES, DnaK/DnaJ, or ready-to-use commercial strains with these optimisations incorporated into the host’s genome, has also been shown to enhance folding and increase the production of active large biosynthetic enzymes [76,78,79]. The example of the anticancer agent epothilone C produced by *Sorangium cellulosum* So ce90 shows that optimising pathway expression often involves a combination of these different strategies to overcome hampered initial attempts by means of low expression levels and limited solubility [79]. The successful generation of this NP is a result of a low expression temperature, promoter engineering, and GroES/EL chaperone co-expression, which helped to improve solubility, and codon optimisation using synthetic genes and three expression plasmids supported the initially low expression of the largest 21.8 kb *epoD*, guaranteeing an efficient biosynthesis of the final NP.

As previously discussed, the size, modular, and repetitive architecture of NRPS/PKS pathways remains a major practical challenge for heterologous expression. However, the inherent modularity can also be exploited by replacement with other systems to either optimise production or produce unnatural analogues. Several positions enabling efficient modular exchange have been developed, such as eXchange units (XUs) [80], eXchange units between thiolation domains (XUTs) [81,82], and eXchange units condensation domain (XUCs) [83]. The XU strategy represents an alternative to module-based NRPS engineering by using smaller, functionally coherent building blocks comprising A-PCP-C or A-PCP-C/E domains. XUs are recombined at conserved C-A linker regions while preserving the substrate specificity of the downstream condensation domain. For example, the exchange of Phe-specific XUs in the NRPS responsible for ambactin biosynthesis resulted in almost no loss in productivity [80]. The limitations regarding the described downstream C-domain specificities are addressed by the XUC strategy, which describes a new fusion point and uses direct assembly within the C domain [83]. Inspired by evolutionary principles, the XUT approach enables precise module exchange in NRPS/PKS systems with excision sites located within the thiolation (PCP/ACP) domains, facilitating seamless recombination while maintaining catalytic activity and thereby overcoming structural constraints to expand chemical diversity. Using this strategy, a novel derivative of the NRP/PK syrbactin was produced in *E. coli* and shown to maintain inhibition of yeast, human constitutive, and immunoproteasome, compared with the natural NP [84]. These approaches help to achieve stable and efficient expression and create suitable avenues for future optimisation of NP production as well as structural diversification.

Beyond folding, post-translational modifications are essential for NRPS and PKS activity. PCP and ACP domains must be converted from their inactive *apo* forms to their active *holo* forms by a phosphopantetheinyltransferase (PPTase) [85]. *E. coli*'s native PPTase AcpS often displays limited activity toward non-native carrier proteins [85]. To overcome this limitation, two *E. coli* strains were generated: *E. coli* GB05-MtaA, which harbours the MtaA PPTase from *Stigmatella aurantiaca* DW4/3-1, and *E. coli* BAP1, which harbours a genomically integrated copy of the Sfp PPTase from *B. subtilis*, as well as several other modifications to enhance biosynthesis of PKS BGCs [86,87]. A comparative study using the microcystin BGC demonstrated that Sfp outperformed several alternative PPTases [88]. A similar study on the applicability of different PPTases revealed their favourability of respective cognate PCP/ACP substrates, implying the use of PPTases cognate to the native host [89]. Overall *E. coli* BAP1 has become a common host when dealing with NRPS/PKS BGCs from a variety of organisms, underlining its broad spectrum of applicability, such as for the antibiotic erythromycin from *Saccharopolyspora erythraea* NRRL 23338/DSM 40517 [90], bilothiazoles [73], bacillamide D [69], radiosumin [67], and hapalosin [65], amongst others.

Type II thioesterases (TEIIs) function as proofreading enzymes to remove aberrant acyl groups from stalled carrier proteins, effectively removing blockages from assembly lines and improving product titres. Deletion of the gene encoding for TEII in the pikromycin pathway of *Streptomyces venezuelae* ATCC 15439 reduced the NP production to 5% [91,92], while disruption of the TEII gene in *Streptomyces fradiae* T59235 similarly decreased tylosin production to 10% [93], highlighting their essential role in efficient biosynthesis. In the study of 6-dEB, the aglycone of erythromycin, the use of the BGC’s TEII was applied as one strategy of the NP production optimisation, increasing the final titre from 100 to 180 mg/L [92,94,95]. Accordingly, co-expression of a cognate or compatible TEII should be considered a standard design element in heterologous expression constructs. Incorporating TEII-encoding genes directly into expression plasmids or ensuring their controlled co-expression can significantly improve pathway robustness and product yield.

Co-expression of NRPSs with cognate or non-cognate MbtH-like proteins (MLPs) can resolve issues with inactive or insoluble expression products. Multiple studies indicate that they play important roles in NP biosynthesis by acting as a facilitator or chaperone [96,97]. Their characteristic flat, double-sided arrowhead structure suggests two distinct interaction surfaces. One face is conserved and interacts with A or PCP domains, whereas the opposite face is more variable and likely mediates system-specific protein interactions. This functional divergence is supported by phylogenetic analyses indicating that many MLPs are paralogs rather than orthologs [96]. In nocardicin A biosynthesis from *Nocardia uniformis* subsp. *tsuyamanensis* ATCC 21806, three of five A domains required the MLP NocI to be active [98]. The same is true for pacidamycin from *Streptomyces coeruleorubidus* NRRL 18370, as PacL showed no adenylation activity unless combined with the MLP PacJ [99]. In *Streptomyces coelicolor* M145, two genes (*cdaX* and *cchK*) encode MLPs, yet only one is required for production of coelichelin and the calcium-dependent antibiotic, demonstrating that both MLPs can substitute for each other, indicating cross-compatibility between both NP pathways [100]. Additional heterologous cross-complementation effects were observed in another pacidamycin study, as heterologous MLPs from kutzneride, glidobactin, vicibactin, and enterobactin pathways could functionally substitute the native PacJ to varying degrees, establishing that MLPs are not always strictly specific to the native organism [101].

### Metabolic layer: precursor and cofactor availability

Even when biosynthetic enzymes are correctly expressed and post-translationally modified, NP formation in *E. coli* may be frequently constrained by insufficient precursor and cofactor availability [102]. NRPS and PKS pathways impose high metabolic demands, requiring proteogenic or non-proteinogenic amino acid building blocks, acyl-CoA extender units, ATP, NAD(P)H, *S*-adenosylmethionine, and specialised cofactors that are often absent, poorly supplied, or tightly regulated in *E. coli*. Similar constraints apply to pathways requiring precursors or cofactors not produced by *E. coli*; for example, *E. coli* lacks endogenous vitamin B_12_ biosynthesis and uptake systems, necessitating external supplementation or helper plasmids (e.g., pBAD42-*btuCEDFB*) for B_12_-dependent enzymes [103].

Metabolic engineering strategies can be applied to overcome these limitations, as exemplified by the heterologous production of erythromycin’s precursor 6-dEB [87]. Here, *E. coli* BAP1 was equipped with TEII in addition to extensive metabolic rewiring to ensure adequate precursor supply. Introduction of a propionyl-CoA carboxylase enabled biosynthesis of (2S)-methylmalonyl-CoA [104], deletion of the competing propionyl-CoA:succinyl-CoA transferase (*ygfH*) prevented substrate loss [105], and overexpression of *atoC* enhanced propionate uptake [106]. Additional optimisation included expression of an *S*-adenosylmethionine synthetase [107]. Production of the final NP erythromycin was achieved using bioreactor cultivation combined with *ygfH* deletion in BAP1 production strains. Introduction of pathways for methylmalonyl-CoA biosynthesis has also been widely applied, using succinyl-CoA from the citric acid cycle as a precursor *via* methylmalonyl-CoA mutase and epimerase [108], with 6-dEB production serving as a proof-of-concept. Moreover, deleting competing metabolic routes, enhancing precursor uptake through transporter engineering, or supplying missing cofactors represent effective approaches. Another example is the engineered strain *E. coli* BWH19 created to enable *de novo* production of l-homophenylalanine [109]. As efficient l-homophenylalanine biosynthesis requires sufficient precursor l-phenylalanine supply, which is limited by feedback inhibition of *aroG* and *pheA*, BWH19 was constructed by integrating feedback-resistant variants of these genes additionally with a deletion of *feaB* and *tyrA* to eliminate the competing l-tyrosine biosynthetic pathway. This was followed by introducing an optimised hybrid l-homophenylalanine BGC on a medium-copy plasmid and resulted in production titres of 1.41 g/L of l-homophenylalanine. Collectively, these interventions highlight the remarkable flexibility of *E. coli* as an engineered host, while also emphasising its native metabolic insufficiency for complex NP biosynthesis.

The ability of NRPS and PKS systems to incorporate non-proteinogenic and otherwise unusual building blocks is a key driver of NP structural diversity. Precursor feeding experiments are a simple yet powerful diagnostic tool to identify metabolic bottlenecks, since several of these important and required building blocks are not native to *E. coli* and therefore require exogenous supplementation to support efficient heterologous pathway activity. In this sense, substrate prediction tools such as PARAS can be helpful to guide potential substrate feeding strategies. Salicylic acid feeding was essential for the production of the siderophore yersiniabactin originating from *Yersinia pestis* CO92 [110], while epothilone C biosynthesis relied on exogenous supply of a synthetic *N*-acetylcysteamine (SNAC) thioester [79]. Beyond enabling pathway completion, precursor feeding can also be exploited to probe substrate promiscuity and diversify product portfolios, as demonstrated for bilothiazole biosynthesis using alternative salicylic and benzoic acid derivatives as starter units [73]. Collectively, these examples emphasise that addressing the metabolic limitations of *E. coli*—through precursor feeding, pathway rewiring, and cofactor provisioning—is often a decisive factor for successful heterologous NP production (Figure 2).

## Extending the functional limits of *E. coli* and complementary strategies

Despite the application of established genetic, protein, and metabolic optimisation strategies in *E. coli*, heterologous expression of NP BGCs does not always result in successful biosynthesis. Emerging approaches in synthetic biology including AI-guided enzyme design and cell-free expression systems offer complementary strategies to overcome functional limitations. In addition, alternative hosts such as cyanobacteria or metabolically specialised non-model organisms can provide more suitable cellular environments for pathways of interest, particularly when phylogenetic compatibility or native metabolic features are critical. While no single host represents a universal solution, expanding the available toolkit beyond *E. coli* remains an important consideration when conventional optimisation strategies in this host organism fail.

Recent advances in synthetic biology are further expanding the engineering of NRPS/PKS systems beyond the classical optimisation strategies discussed above. AI-assisted enzyme design and generative modelling are emerging as powerful tools to optimise domain compatibility, stability, and catalytic efficiency, enabling the construction of functional hybrid assembly lines with enhanced yields [111]. For example, pretrained generative AI models (ESM3, ProteinMPNN, and EvoDiff) were integrated with iterative design–build–test–learn cycles to generate *de novo* PCP domains, which were evaluated in 578 engineered NRPS variants spanning minimal, full-length, and hybrid assembly lines *in vivo*. The AI-designed domains enabled functional recombination at domain interfaces, improved soluble expression and stability, and increased product titres by up to ∼3-fold compared with native counterparts. This progression from the already mentioned XUT approach demonstrates the strong potential of generative design to overcome domain incompatibility and enhance the performance of complex biosynthetic assembly lines [111].

Complementary to cell-based strategies, cell-free systems are gaining attention as alternative production platforms [112,113]. Cell-free protein expression (CFE), including cell-free protein synthesis (CFPS), is based on *in vitro* transcription–translation systems that utilise either crude cell extracts or purified components to synthesise proteins outside living cells. It enables rapid, high-throughput expression independent of cellular viability, thereby overcoming limitations such as metabolic burden, toxicity, and complex regulation [112]. CFPS and CFE allow precise control over reaction conditions, direct manipulation of substrates and cofactors, and rapid prototyping of biosynthetic pathways. Valinomycin production provides a representative example for the application of cell-free systems in comparison with *E. coli*-based heterologous expression. *In vivo* expression of the NRPS genes *vlm1* and *vlm2* in *E. coli* achieved titres of ∼1 mg/L following extensive bioprocess optimisation [114,115]. Initial *E. coli* lysate-based CFPS enabled the co-expression of Vlm1 and Vlm2, together with Sfp, in a one-pot reaction, yielding ∼9.8 μg/L. Inclusion of a TEII further increased titres to ∼37 μg/L [116,117]. Subsequent integration of CFPS with cell-free metabolic engineering (CFME), using lysates enriched in Vlm1 and Vlm2 and optimised reaction conditions, significantly enhanced production to ∼29.3 mg/L [118–120]. This progression highlights the capacity of cell-free systems to support complex NRPS biosynthesis and demonstrates their potential to achieve and even exceed *in vivo* production levels while offering increased control over pathway components and reaction conditions [112]. Although challenges such as scalability and cofactor regeneration remain, cell-free approaches represent a powerful complement to *in vivo* expression, particularly for complex or toxic pathways [113].

When such strategies fail to restore pathway activity, transitioning to alternative hosts represents a justified and complementary progression rather than an exploratory first step or a strict last resort. Cyanobacteria have emerged as promising hosts for photosynthetically driven NP biosynthesis [46,121–123]. These hosts offer the unique advantage of coupling secondary metabolism to light-driven carbon fixation as well as expressing cyanobacterial BGCs due to their closer phylogenetic relationship and compatible cellular machinery, although slower growth and limited genetic accessibility remain challenges [46,121]. This is illustrated by the non-NRPS/PKS ambigol pathway from *Symphyonema bifilamentata* sp. nov. 97.28 (formerly *Fischerella ambigua* 108b), which could not be functionally expressed in *E. coli* BAP1 despite confirmed transcription, likely due to limitations in protein folding or enzyme functionality, but was successfully expressed in *Synechococcus elongatus* PCC 7942 [124]. Lyngbyatoxin is a fascinating example of heterologously expressing a BGC across various hosts. Expression of the pathway failed in *Streptomyces* (hypothesised to be transcription failure of *ltxA*) but was successful in *E. coli* GB05-MtaA, along with the biosynthetic intermediate indolactam V as the major compound. However, the cyanobacterial host *Anabaena* sp. PCC 7120 was able to produce lyngbyatoxin as the major compound with titres comparable to the native producer [77,121,125]. These examples emphasise the need for systematic comparison of BGC expression across hosts and that host-specific factors, such as cofactor availability and regulatory compatibility, can critically influence NP expression.

Metabolically versatile bacteria, such as *P. putida*, have gained attention as heterologous expression hosts with high solvent tolerance and a growing synthetic biology toolkit, making them well suited for PKS and NRPS/PKS-hybrid pathways imposing high metabolic burden [126,71]. *Burkholderia* [127], *Photorhabdus laumondii* TTO1 [47], *Xenorhabdus* sp. TS4 [47], *Salinispora tropica* CNB-440 [128], and the facultative anaerobe *Streptococcus mutans* UA159 [129], amongst other native or near-native producers, provide additional options. Endogenous expression in these non-model organisms can leverage evolutionary adaptation, reducing the need for extensive pathway refactoring. Expanding the heterologous host range benefits from expanded precursor pools, robust stress tolerance, as well as intrinsic specialised metabolic and resistance mechanisms [71]. Multi-omics technologies increasingly allow systematic identification, characterisation, and engineering of such hosts [71]. However, challenges including genetic accessibility, scalability, and process standardisation remain.

## Conclusion

Looking forward, *E. coli* will remain the central and most accessible platform for NP expression, driven by its growth rate, unparalleled genetic toolbox, experimental tractability, and rapidly expanding repertoire of engineered strains and helper plasmids. Heterologous expression in *E. coli* is best approached as a systematic diagnostic process rather than a single engineering task. Iterative optimisation across genetic, protein and metabolic layers has transformed initial failures into successful production systems. Future developments may involve the engineering of increasingly specialised expression strains ever more tailored towards NP biosynthesis. Such next-generation strains could integrate enhanced folding capacity, expanded and balanced precursor supply, improved cofactor regeneration, and reduced proteolysis, while maintaining genetic stability. Modular strain design, analogous to modular plasmid systems, may further streamline pathway optimisation, while alternative microbial hosts provide important alternatives for particularly recalcitrant BGCs. Equipped with the growing toolbox of optimisation strategies outlined here, *E. coli* remains not only a workhorse but also a valuable guide in navigating the broader landscape of microbial NP biosynthesis.

## Summary

- *E. coli* represents the central and most accessible heterologous host for recombinant NP biosynthesis, combining rapid growth, experimental tractability, and an extensive toolbox for genetic and metabolic engineering.
- Expression of complete NP pathways in *E. coli* requires strategies beyond single-protein overexpression, demanding balanced control of gene expression, folding, post-translational modification, and metabolic flux.
- Systematic, iterative optimisation in *E. coli* can overcome most pathway-related bottlenecks, enabling successful reconstitution of complex NRPS and PKS systems.
- When comprehensive optimisation and troubleshooting in *E. coli* remains unsuccessful, non-model organisms offer complementary alternative microbial chassis, particularly for pathways incompatible with *E. coli*'s regulatory or metabolic framework.

## Open Access

Open access for this article was enabled by the participation of Saarland University in an all-inclusive Read & Publish agreement with Portland Press and the Biochemical Society under an agreement with the TIB German Alliance.

## Abbreviations

6-dEB: 6-deoxyerythronolide B
A domain: adenylation domain
ACP: acyl carrier protein
AI: artificial intelligence
AT domain: acyltransferase domain
ATP: adenosine triphosphate
BGC: biosynthetic gene cluster
C domain: condensation domain
CFE: cell-free expression
CFME: cell-free metabolic engineering
CFPS: Cell-free protein synthesis
CoA: Coenzyme A
CRISPR: Clustered regularly interspaced short palindromic repeats
Cy domain: cyclisation domain
DH: dehydratase
DiPaC: Direct Pathway Cloning
E domain: epimerisation domain
ER: enoyl reductase
IPTG: isopropyl β-d-1-thiogalactopyranoside
KR: ketoreductase
KS: ketosynthase
LCHR: linear-circular homologous recombination
LLHR: linear-linear homologous recombination
MLP: MbtH-like protein
MT: methyltransferase
NAD(P)H: nicotinamide adenine dinucleotide (phosphate)
NP: natural product
NRP: non-ribosomal peptide
NRPS: non-ribosomal peptide synthetase
OSMAC: One Strain Many Compounds
PCP: peptide carrier protein
PK: polyketide
PKS: polyketide synthase
PPTase: phosphopantetheinyl transferase
RBS: ribosome-binding site
RiPP: ribosomally synthesised and post-translationally modified peptides
SNAC: synthetic *N*-acetylcysteamine
TAR: transformation-associated recombination
TE: thioesterase
TEII: type II thioesterase
XU: eXchange Unit
XUC: eXchange Unit condensation domain
XUT: eXchange Unit between PCP domains
